# Disentangling the initiation from the response in joint attention: an eye-tracking study in toddlers with autism spectrum disorders

**DOI:** 10.1038/tp.2016.75

**Published:** 2016-05-17

**Authors:** L Billeci, A Narzisi, G Campatelli, G Crifaci, S Calderoni, A Gagliano, C Calzone, C Colombi, G Pioggia, F Muratori, ALERT group, Rossella Raso, Liliana Ruta, Ilaria Rossi, Agnese Ballarani, Francesca Fulceri, Alessandra Darini, Emilia Maroscia, Caterina Lattarulo, Gaetano Tortorella, Rosamaria Siracusano, Valentina Comminiello

**Affiliations:** 1 Institute of Clinical Physiology, National Research Council of Italy (CNR), Pisa, Italy; 2 Department of Clinical and Experimental Medicine, University of Pisa, Pisa, Italy; 3 Department of Developmental Neuroscience, IRCCS Stella Maris Foundation, Pisa, Italy; 4 Division of Child Neurology and Psychiatry, University of Messina, Messina, Italy; 5 Child Neuropsychiatry Unit, Hospital ‘Madonna delle Grazie', Matera, Italy; 6 Department of Psychiatry, University of Michigan, Ann Arbor, MI, USA; 7 Institute of Applied Sciences and Intelligent Systems “Eduardo Caianiello” (ISASI), National Research Council of Italy (CNR), Messina, Italy

## Abstract

Joint attention (JA), whose deficit is an early risk marker for autism spectrum disorder (ASD), has two dimensions: (1) responding to JA and (2) initiating JA. Eye-tracking technology has largely been used to investigate responding JA, but rarely to study initiating JA especially in young children with ASD. The aim of this study was to describe the differences in the visual patterns of toddlers with ASD and those with typical development (TD) during both responding JA and initiating JA tasks. Eye-tracking technology was used to monitor the gaze of 17 children with ASD and 15 age-matched children with TD during the presentation of short video sequences involving one responding JA and two initiating JA tasks (initiating JA-1 and initiating JA-2). Gaze accuracy, transitions and fixations were analyzed. No differences were found in the responding JA task between children with ASD and those with TD, whereas, in the initiating JA tasks, different patterns of fixation and transitions were shown between the groups. These results suggest that children with ASD and those with TD show different visual patterns when they are expected to initiate joint attention but not when they respond to joint attention. We hypothesized that differences in transitions and fixations are linked to ASD impairments in visual disengagement from face, in global scanning of the scene and in the ability to anticipate object's action.

## Introduction

Joint attention (JA) is described as the ability to coordinate visual attention with another person and then shift the gaze toward an object or event^1^ and does not require the gazer to be aware of the follower's reaction.^2^ Literature reports two main components of JA: (1) response to JA and (2) initiation of JA. Responding JA is described as the ability to shift visual attention following other's social cues such as gaze or pointing, whereas initiating JA is the ability to direct another person's attention through gaze or gestures with the aim of sharing an experience.^3^ Responding JA and initiating JA are considered as two interrelated aspects of JA, emerging at different times during development.^4^ Responding JA usually develops between 6 and 9 months of age, whereas initiating JA starts approximately at 9 months of age with great variability across individuals.^4, 5, 6^ Most studies involving joint attention have focused on responding JA,^6, 7, 8, 9, 10, 11^ whereas only a limited number of investigations have evaluated initiating JA.^12, 13, 14, 15^

Charman,^16^ in his review, has concluded that JA impairment is one of the more precocious and consistent early sign of autism spectrum disorder (ASD) and these impairments correlate with social-communication impairments, as well as language delays. It has been described that difficulties in responding JA emerge during the first year of life and that become progressively evident during the second year when difficulties in initiating JA become a hallmark of ASD.^17^ For this reason, a multi-item joint attention factor is considered in Modules 1 and 2 of the Autism Diagnostic Observation Schedule (ADOS).^18^

Recently, eye-tracking has largely been used to investigate visual patterns during JA tasks in ASD. Indeed, eye-tracking assessments of JA may provide more precise spatial and temporal information than behavioral coding.^19^ Different studies have been focused on gaze-following tasks to explore responding JA ability in young children;^6, 10, 11, 20^ however, contradictory results have been found. For example, Bedford *et al.*,^10^ using video sequences in which a female model between two objects alternatively turned her head towards one of the objects, did not find differences in terms of gaze accuracy (that is shifting gaze to the attended rather than the unattended object) between children with ASD and those with typical development (TD). Chawarska *et al.*,^6^ using photographs of faces processed to reproduce the effect of gaze shift, found similar results but evidenced that difficulties in gaze following emerge along with the severity of the socio-communicative impairment. Differently, Falck-Ytter *et al.*,^11^ using video sequences with a model looking, pointing or both looking and pointing toward an object, found that preschoolers with ASD performed fewer and slower correct gaze shifts compared with preschoolers with TD. Differences among the type of social stimuli (that is, static faces on the screen vs more dynamic and naturalistic videos) used in these eye-tracking studies could be responsible for the contradictory results.

It is worth mentioning that social stimuli in an eye-tracking scenario could elicit a different level of attention than that in a real-life situation. For example, in a responding task, we cannot be sure that children follow a model's gaze in the video in the same way that they would follow someone's gaze in real life. The problem of social stimuli is even more challenging in an initiating JA task where children could shift attention to the model just for monitoring the scene than to create an initiating joint attention situation.

Aware of these video-based eye-tracking limitations, and following some recent literature on initiating JA,^12, 13, 14, 15^ we attempt to translate clinical studies on both responding JA and initiating JA, into an eye-tracking scenario. To the best of our knowledge, this is the first eye-tracking study of initiating JA involving young children with ASD. The aim of our study was to describe the visual patterns of toddlers with ASD and TD during tasks eliciting responding JA, as well as tasks eliciting initiating JA. Two different initiating JA tasks were developed to investigate initiating JA abilities: via a predictable and unpredictable event. We hypothesized that differences between children with ASD and children with TD would increase from the responding JA task to initiating JA task.^21^

## Materials and methods

### Participants

A group of 17 children with ASD and a group of 15 children with TD between 18 and 30 months of age were enrolled in the study. The ASD group was recruited in three different Institutions: the Autism Unit of IRCCS Stella Maris Foundation of Pisa, the Division of Child Neuropsychiatry of the University Hospital of Messina and the Hospital of Matera. The clinical diagnosis of ASD was established according to DSM-5 criteria^22^ and confirmed using algorithm cutoffs on the ADOS-2,^23^ which was administered by ADOS research reliable examiners. In addition, the parents of the children with ASD completed the M-CHAT.^24^ The exclusion criteria were as follows: (a) neurological syndromes or focal neurological signs; (b) significant sensory impairment; (c) anamnesis of birth asphyxia, premature birth, head injury or epilepsy; (d) use of any psychotropic medication; and (e) potential secondary causes of ASD determined by high-resolution karyotyping, DNA analysis of Fragile-X or screening tests for inborn errors of metabolism.

The participants with TD were recruited from daycares in the Pisa, Messina and Matera metropolitan areas. All children (ASD and TD) received a nonverbal developmental evaluation through the administration of the performance subscale of the Griffiths Mental Developmental Scales. TD was also confirmed by a Child Behavior Check List (CBCL) Total score under the borderline/clinical range that was considered mandatory for the inclusion of TD children (Table 1).

Some items from the ADOS-2 and the M-CHAT were chosen as concomitant clinical measures of joint attention. ADOS-2 items belonging to the joint attention factor^25^ were selected: pointing; response to joint attention; gesturing; showing; initiation joint attention and unusual eye contact. The M-CHAT item n°7 (Does your child ever use his/her index finger to point, to indicate interest in something?) was also selected as a parent-reported measure of joint attention.

All parents provided written informed consent including permission to use the video recordings for scientific reasons. The experimental procedures and the informed consent were approved by the ethics committee of the IRCCS Stella Maris Foundation (Pisa, Calambrone, Italy).

### Procedure

Toddlers' gaze was recorded by means of the SMI Eye Tracking device provided by SensoMotoric Instruments (Teltow, Germany), with a sample rate of 120 Hz and accuracy better than 1 degree of visual angle. The eye-tracker records the data of both eyes from the reflection of near-infrared light on the cornea and pupil. It was positioned in front of the subject just below a 22-inch flat screen monitor where the stimuli were presented using SMI Experiment Center Software. The distance from the screen and the inclination angle of the system were adjusted for each toddler to obtain a good tracking of his/her eyes. The placement suggestions provided by SMI iViewX Software were used for the correct positioning of the eye-tracker. The toddlers sat on a child chair to limit movements. The distance of the subjects from the screen was approximately 50 cm. Before starting the experimental task, a five-point calibration sequence was run. A cartoon was chosen as calibration point to increase the toddlers' attention to the screen. The calibration was repeated until the deviation from the known calibration target for both the x and y components was below 2°. The accuracy of calibration between the two groups was calculated as the root mean square value of the deviation of the x and y components. The root mean square was not significantly different between the two groups (ASD=1.2±0.6, TD=1.2±0.7, *P*=0.7).

### Stimuli

A video demo, showing and describing the experimental setting and the three tasks, is provided in the Supplementary Materials.

**Figure 1:**
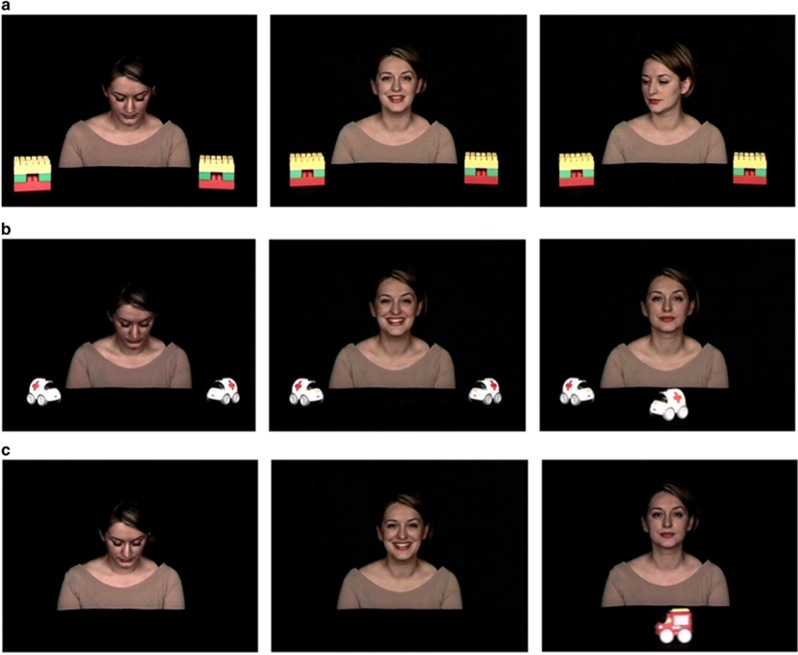
Screen shots of the videos of the different tasks. (**a**) Responding to joint attention task (responding JA), (**b**) initiating joint attention with two objects on the scene (initiating JA-1), (**c**) initiating joint attention with one object appearing from outside of the scene (initiating JA-2). The three tasks are divided into the three different phases: looking down, interactive, joint attention (JA).

The experiment consisted of three tasks (one for responding JA and, following the distinction between predictable and unpredictable event, two tasks for initiating JA), each composed of three segments (Figure 1): looking down (2 s), interaction (2 s) and joint attention (4 s for responding JA and 7 s for initiating JA-1 and initiating JA-2).

Because of the focus of the paper, we describe here only the JA segment of the three tasks. In the JA segment of the responding JA task, the model turns her head toward one of the two blocks (target object) and then fixates that block; the other block on the opposite part of the scene is by convention the ‘non-target object'. In the JA segment of the first initiating JA task (initiating JA-1), one of the two toy cars (target object) starts moving on the screen toward the other car (non-target object) until it reaches approximately the center of the screen while the model keeps a direct gaze towards the camera maintaining a neutral, impassive facial expression. In the JA segment of the second initiating JA task (initiating JA-2), a toy truck (target object) appears unexpectedly from outside of the scene and crosses the screen toward the opposite side while the model keeps a direct gaze towards the camera maintaining a neutral, impassive facial expression. This impassive, non-interactive facial expression was chosen to ensure that child's eye gaze toward the model's face was not in response to a solicitation from the model.

All the trails were presented in a block design paradigm. Each block consisted of four repetitions of one task and the sequence of the blocks was always: responding JA, initiating JA-1, initiating JA-2. A total of 12 trials were presented to each child. Each trial was preceded by a colorful ‘attention-getter' that was displayed at the center of the screen until the toddler looked at it for at least 500 ms. This phase was necessary for re-centering the eyes before the beginning of the trial. Once attention was secured, the pre-recorded video replaced the attention-getter. Some trials were excluded on the basis of the criteria adopted by Bedford *et al.*^10^ adapted to our tasks. The trial exclusion criteria were as follows: (1) no looking at the face during the ‘interactive' segment, which can be considered as a prerequisite for joint attention behavior;^26^ and (2) looking away from the computer screen for the entire ‘joint attention' phase. Repeated-measures analysis of variance revealed that there was no significant effect of task (F=2.6, *P*=0.8) or group × task (F=0.01, *P*=0.9) on number of usable trials. The mean number of usable trials was 10.8±1.5 for the ASD group and 11.1±1.7 for the TD group.

### Measures and data analysis

Considering the focus of the paper and the JA construct, the measures were computed only for the JA segment of the three tasks. Normalized gaze or object following the accuracy, transitions and fixations on areas of interest (AOIs) were considered as measures for analysis (see Supplementary Table S1 for the exact definition of these measures). Gaze or object following accuracy refers to the difference between frequency of first looks at the target object (that is the object looked by the actress or the moving object) and frequency of first look to the non-target object.

The normalized gaze-following accuracy for the responding JA task and the normalized object-following accuracy for the initiating JA-1 were computed as the difference of frequency of first looks at the target object and the frequency of first looks at the non-target object and dividing this difference by the number of trials in which the child looked to either objects.^9, 26^ These measures are an index of child's preference for target or non-target object.

Measures referred to transitions were computed by extracting raw data and analyzing them in Matlab (MathWorks, Natick, MA, USA) using homemade scripts. To explore transitions in responding JA, we computed the number of transitions from face to target object and from face to non-target object. Then, a normalized transition score (that is the difference between total number of transitions from face to target object and total number of transitions from face to non-target object, divided by the total number of transitions from face to either objects across trials) was calculated. To explore the alternating looking pattern between face and object in the two initiating JA tasks, we evaluated the number of transitions from face to target object and the number of transitions from target object to face. In initiating JA-1, we also computed the number of transitions from face to non-target object, the number of transitions from non-target object to face and between-objects transitions. Then, for initiating JA-1, the normalized transition score (that is the difference between the total number of transitions from target object to face and the total number of transitions from non-target object to face divided by the total number of transitions from either object to face) was calculated. It should be noted that the normalized scores are divided by total looks to either objects and thus if the total number of transitions increases, the value of the normalized score decreases.

Using SMI BeGaze Software (SensoMotoric Instruments) the following AOIs were selected: model's face, target object, non-target object. To explore the child's engagement with each AOI, fixation duration (FD) within the selected AOIs was analyzed.^26, 27^ To avoid unconscious looking, a fixation threshold of 60 ms was applied to the raw data as already performed in the study by Falck-Ytter *et al.*^20^ on toddlers. FD on a specific AOI was computed as a percentage of the total, which means FD on that AOI relative to the participants' on-trial FD. Because in the responding JA task the aim was to investigate possible differences in ASD or TD performance when children do follow the model's gaze, FD for objects were analyzed only for trials with a congruent first gaze shift.^10, 20^ This kind of analysis was extended to the two initiating JA tasks: to assess the interaction with face after looking at the object, we analyzed FD at face considering only trials with first look at the target object.

### Statistical analyses

Statistical analyses were performed in SPSS (SPSS, Chicago, IL, USA). A comparison between children with ASD and children with TD was performed in terms of demographic, cognitive level and eye-tracking data. The Shapiro–Wilk test was applied to test the normality of the variables.

Given that the two groups were different in terms of Griffiths performance, this measure was used as covariate in all the analyses.

For normal variables, an analysis of covariance test was applied. When a non-parametric test was required, variables and covariate were transformed in ranks and the analysis of covariance on ranks was performed. Levene's test for homogeneity of variances was applied in the analysis of covariance analysis. To correct for multiple comparisons, false discovery rate^28^ was applied using a *q*-value of 0.05. Effect sizes were estimated by partial eta squared (*η*^2^; values between 0.01 and 0.06 are generally considered a small effect, between 0.06 and 0.14 a medium effect and those above 0.14 are regarded as a large effect).

Pearson correlations or Spearman correlations, according to the distribution of the variables, were used to examine correlations amongthe following: (1) comparable eye-tracking measures in initiating JA-1 task and initiating JA-2 (separately within each group); (2) eye-tracking measures and selected items from ADOS-2 and M-CHAT, in the ASD group.

### Code availability

The scripts used for the transitions and transitions score computation are available on request from the first author (LB).

## Results

### Normalized gaze- and object-following accuracy

The two groups were not significantly different in terms of normalizing gaze-following accuracy in responding JA or in normalized object following in initiating JA-1 task. The positive value of these measures indicates that both groups had higher number of first look to the target than to the non-target object.

### Transitions

**Figure 2:**
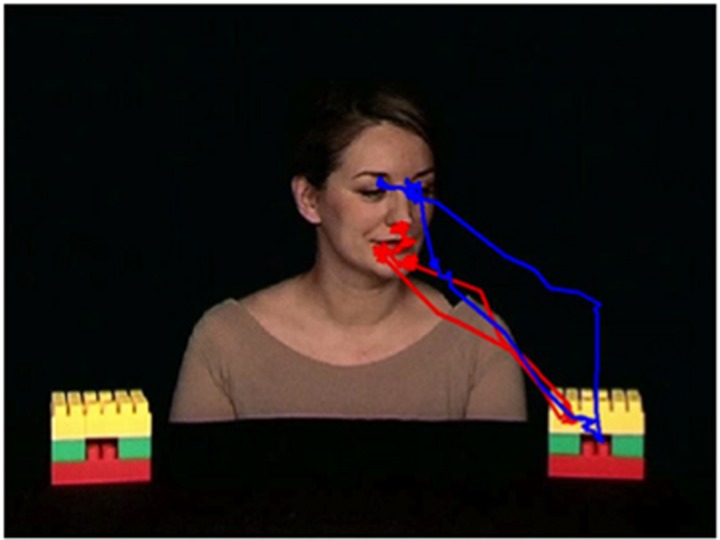
Scan path for a viewer with autism (red trace) and for a viewer with typical development (blue trace), during the ‘joint attention' phase of responding to joint attention task (responding JA).

The results are reported in Table 2. In the responding JA task, the number of transitions from face to both the target object and the non-target object and the normalized transition score were not significantly different between ASD and TD groups. In Figure 2a, scan path for a viewer with ASD and for a viewer with TD during the ‘joint attention' phase of the responding JA task is reported.

**Figure 3:**
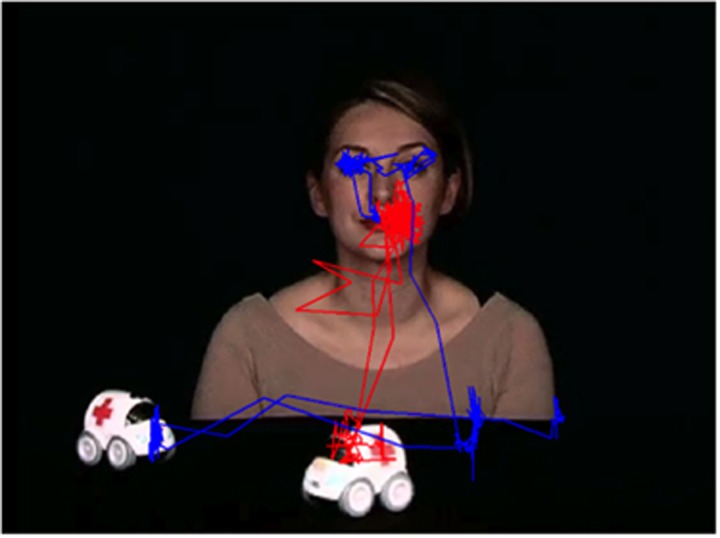
Scan path for a viewer with autism (red trace) and for a viewer with typical development (blue trace), during the ‘joint attention' phase of the task of initiating joint attention with two objects (initiating JA-1).

In initiating JA-1 task, the normalized transition score was significantly higher in ASD compared with TD (*P*=0.02), meaning that ASD group looked more at the target than at the non-target object and had a reduced global number of transitions compared with the TD group. When analyzing transitions separately, TD group showed a higher number of transitions from non-target object to face (*P*=0.02) compared with ASD. As regards the inverse transitions, there was a significant increase of transitions from face to target object in the ASD group (*P*=0.02). Between-objects transitions were higher in the TD group compared with the ASD group (*P*=0.02). In Figure 3a, scan path for a viewer with ASD and for a viewer with TD during the ‘joint attention' phase of the initiating JA-1 task is reported.

**Figure 4:**
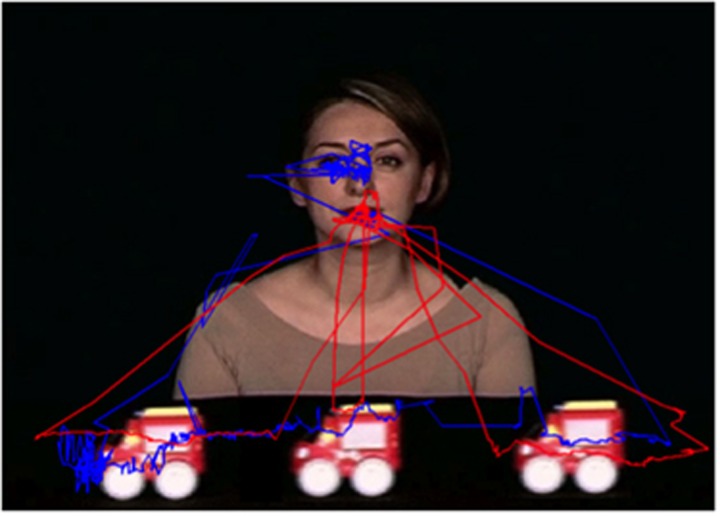
Scan path for a viewer with autism (red trace) and for a viewer with typical development (blue trace), during the ‘joint attention' phase of the second task of the task of initiating joint attention with one object (initiating JA-2).

In initiating JA-2 task, the ASD group showed a higher number of transitions both from target object to face (*P*=0.01) and from face to target object (*P*=0.008). In Figure 4a, scan path for a viewer with ASD and for a viewer with TD during the ‘joint attention' phase of the initiating JA-2 task is reported.

### Fixations

**Figure 5:**
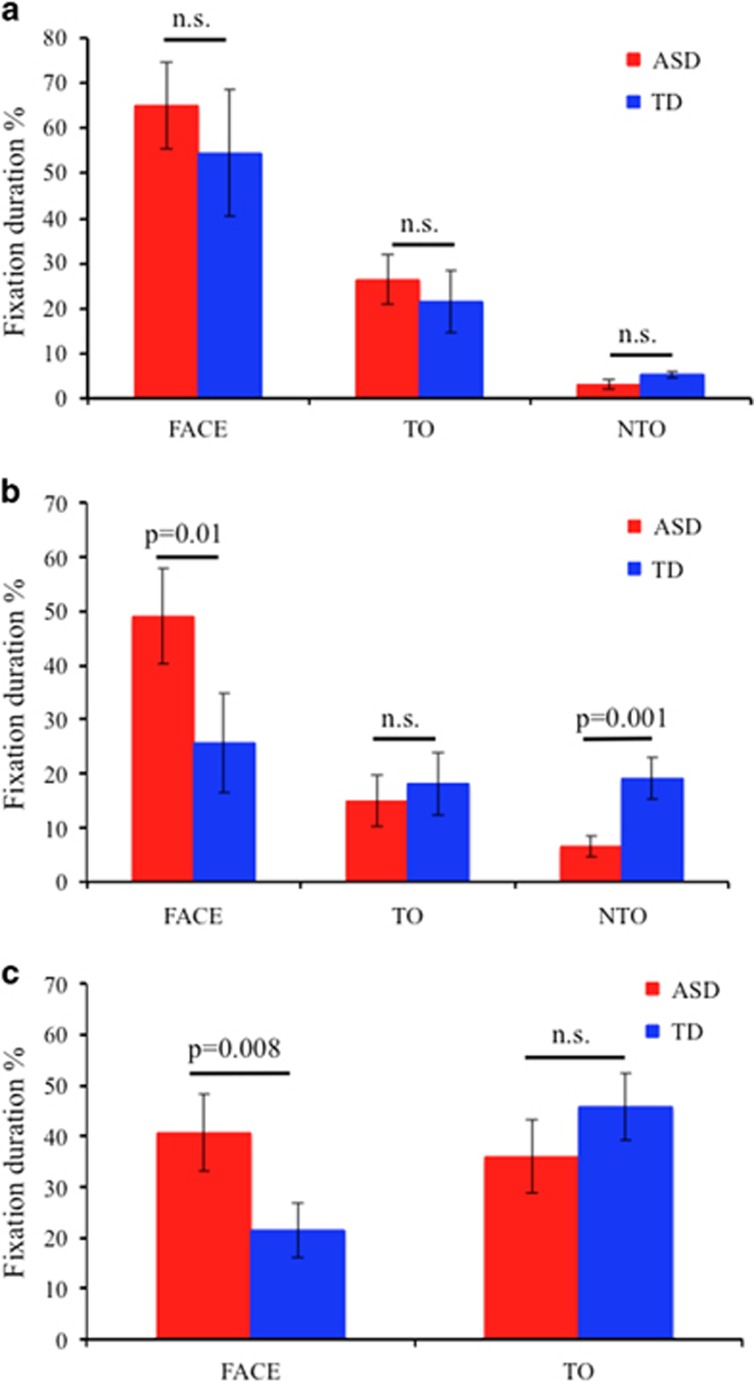
Percent fixation duration in the areas of interest of subjects with autism and typical development (TD) in responding joint attention (JA) (**a**), initiating JA-1 (**b**) and initiating JA-2 (**c**) tasks. Significance are provided for analysis of covariance performed on normally distributed variables. ASD, autism spectrum disorder.

The results are reported in Figure 5. In responding JA task, the two groups did not perform differently in terms of FD for target object, for non-target object and for face (Figure 5a).

In initiating JA-1 task, the TD group had higher FD (*P*=0.001) for the non-target object compared with the ASD group while the ASD group had higher FD (*P*=0.01) for face compared with the TD group (Figure 5b). When the analysis replicated considering only trials in which subjects first looked at the target object, differences remained significant (*P*=0.01).

In initiating JA-2 task, the ASD group had higher FD (*P*=0.01) for face compared with the TD group; considering only trials in which subjects looked first at the target object, the ASD group still have higher FD for face (*P*=0.008; Figure 5c).

### Correlations between comparable eye-tracking measures in initiating JA-1 and initiating JA-2 task

In the ASD group, there was a significant positive correlation between transitions from target object to face in initiating JA-1 and transitions from target object to face in initiating JA-2 (*r*=0.73, *P*=0.001) and between transitions from face to target object in initiating JA-1 and transitions from face to target object in initiating JA-2 (*r*=0.80, *P*<0.001).

In the TD group, there was a significant positive correlation in transitions from face to target object between initiating JA-1 and initiating JA-2 (*r*=0.66, *P*=0.009).

### Correlations between eye-tracking measures and clinical measures in ASD

In the responding JA task, a significant negative correlation was found between transitions from face to target object and both ADOS_A7-Pointing (*r*=−0.57, *P*=0.02) and M-CHAT_item 7 (*r*=−0.55, *P*=0.03). This result indicates that a lower number of transitions is correlated with more difficulties in pointing in real-life situations.

In initiating JA-1, the ADOS_B9 (showing) and M-CHAT_item 7 (pointing) were negatively correlated with transitions from face to non-target object (*r*=−0.63, *P*=0.009 and *r*=−0.64, *P*=0.01, respectively) and with transitions from non-target object to face (*r*=−0.62, *P*=0.01 and *r*=−0.60, *P*=0.02, respectively). This result indicates that a lower number of transitions from and to the non-target object is correlated with more impairments in clinical measures of JA.

In initiating JA-2, there was a significant positive correlation between ADOS_B1 (eye contact) and both transitions from target object to face (*r*=0.54, *P*=0.03) and transitions from face to target object (*r*=0.56, *P*=0.02). This means that a higher number of transitions from target object is correlated with more difficulties in eye contact.

## Discussion

The present eye-tracking study examined the performance of toddlers with ASD to different tasks exploring the two components of JA (responding and initiating). The main findings can be summarized as follows: (a) eye-tracking measures do not show differences between ASD and TD during the responding JA task; (b) in the initiating JA task with an expected event, toddlers with ASD look longer at face and have more transitions from face to the moving object while the TD group show more fixations for the still object and more transitions from this object to model's face; (c) in the initiating JA task with an unpredictable event, toddlers with ASD looked longer to face and more transitions from the object to face.

The lack of differences in responding JA (normalized gaze-following accuracy, fixations in AOI and number of transitions from face to target object) indicates similar abilities in ASD and TD for gaze following and modulation of the focus of visual attention. This finding is in line with an emerging view that gaze following is not impaired in young children with ASD,^10, 20, 29^ while it could be questioned whether this is a specific effect of the experimental design that could not be replicated in a real-life setting.^30^ Nevertheless, transitions from face to target object were negatively correlated with pointing at both ADOS and M-CHAT, indicating that children with more socio-communicative impairments in real life have a tendency to show less shifts towards the target object in our eye-tracking task. A similar finding was described by Chawarska *et al.*^6^

Although in the responding JA task ASD and TD seem to behave similarly, in initiating JA tasks. some differences emerge in terms of transitions and fixations. In particular, our results indicate that (i) children with ASD looked longer to face and had more transitions from target object to face and (ii) TD children were more engaged in the non-target object, had more shifts from non-target object to face and made more transitions between the two objects. Moreover, the higher normalized transition score in children with ASD means that these children, compared with TD, had higher transitions from target object to face than from non-target object to face. Although these findings may appear counterintuitive, several explanations could be given. These explanations go beyond the salience of the moving object for children with ASD. First, it should be observed that the preference for objects is not a hallmark of ASD, but it is dependent on the context, and it can be modulated by the salience of the social stimuli. Similarly, another recent eye-tracking study with preschoolers found that preference for faces presented along with objects is not diminished in children with ASD compared with TD children.^31^ Second, our results could be the expression of specific impairments in visual disengagement.^32^ Initiating JA is a complex process that involves disengaging from an object to look fluently towards the partner's face, followed by disengaging from the partner's face to reorient back towards the attended object.^33^ Recent investigations indicate that 7-month-old infant siblings subsequently diagnosed with ASD showed longer latencies disengaging from central stimuli to look at peripheral ones,^34^ and that this failure could interfere with the development of JA.^35^ According to this view, a positive correlation between faster disengagement and initiating JA in young children with ASD was reported.^36^ Thus, we could suggest that the higher fixation duration at face in our children with ASD could be linked to the impairment in disengaging from face.

Third, the lower attention to non-target object in ASD could be related to a deficit in divided attention, which might impair the ability to track more than one object on the scene.^37, 38, 39^ The importance of scanning the global scenario in a JA task is confirmed by the negative correlation between number of transitions and clinical measure of JA (showing at ADOS, pointing at M-CHAT), that means that a lower number of transitions correlates with more problems in JA behaviors. Another hypothesis for the lower attention to the still non-target object could be the difficulties in anticipation that have been described as one of the strongest indicators of an ASD.^40, 41^ We may propose that TD children have higher attention to the non-target object because they, differently from ASD, foresee its possible movement.

In the second task for initiating JA task, the child was confronted to an unexpected event (the truck crossing the screen) and to an adult not interested in this event. Our hypothesis was that this scenario should have been able to push the child to look at the actress's face for inviting her to focus the attention on the new event. Our findings show that children with ASD have more transitions from object to face and vice versa and more attention to face. This pattern would seem to indicate a JA behavior in ASD;^42^ thus, we could hypothesize that children with ASD do not suffer from across-the-board disruptions of JA, and that a non-predictable situation in an eye-tracking scenario could elicit skills that are less expressed in real-life situations. Otherwise, we may suppose that the exceeding looking at face in children with ASD could be linked to the sudden appearance of the little truck. In fact, even if the truck is attractive for children with ASD because of its mechanical movement,^43^ it could represent a challenge for the insistence on sameness of these children;^44, 45^ as a consequence, they come back to the ‘known and predictable' face more frequently than TD children.

Thus, the two tasks for initiating JA allow supporting the hypothesis that social attention is expressed in a different manner in young children with ASD. Indeed, in both tasks, these children showed a higher number of transitions from target object to face. Moreover, the number of transitions from target object to face in the second initiating JA task was positively correlated with the item ‘eye-contact' at ADOS. To summarize, we may conclude that an excessively high number of transitions, as we found in both initiating JA tasks, could not be totally appropriate for the development of initiating JA.

This study has showed that initiating JA can be studied using eye-tracking paradigms and that these kinds of assessments can give new insights regarding the atypical development of JA in the very early stages of ASD. Moreover, our results suggest not only the importance of disentangling initiating JA from responding JA, but also the opportunity of disentangling the two different types of initiating JA. In fact, while confirming the clinical suggestion that initiating JA is more able to detect differences between ASD and TD children than responding JA,^46, 47^ our study shows that the two tasks for initiating JA are more suitable to explore the underlying reasons of JA difficulties shown by children with ASD. Thus, it could represent a starting point toward a better description of the underpinnings of the developmental trajectory for initiating JA that differs fundamentally from that of non-autistic children.

### Limitations

A potential limitation is the absence of a control group with a developmental quotient similar to that of the ASD group. The comparison with a TD group could prevent our result to be considered specific of ASD, and could be questioned whether the group effects reflect differences specifically due to ASD. Nevertheless, this limitation is reduced by the use of the nonverbal development quotient as a covariate in all between-subject comparisons, making our results likely independent from intellectual development.

Second, we have to consider that in the video-based eye-tracking scenario, we get a partial evaluation of initiating JA as compared with a real-life situation.^48^ In fact, we have evaluated initiating JA in an experimental context in which a child's eye gaze is expected to search for an adult impassive face. In future, a more ecological paradigm, where the realization of child's initiating JA is encouraged by an adult's expression of a positive affection, would contribute to an even more accurate evaluation of initiating JA.

Third, the number of cases in the two samples was consistent for a classical univariate comparison but it is too small to attempt to establish whether some significant variables can be used to distinguish between ASD and TD. For this aim, a multivariable mathematical model could be implemented in the future on a larger sample to describe the predictive accuracy of some variables.

## Figures and Tables

**Table 1:** Demographic and clinical characteristics of the ASD and TD groups of children

	*ASD (*N*=17)*	*TD (*N*=15)*	*Comparison coefficients*	P*-value*
	*M (s.d.)*	*M (s.d.)*		
Age (months)	26±4.2	26.1±3.9	*t* (30)=1.06	0.32
Gender: M, F	14, 3	13, 2	*χ*^2^=0.23	0.71
ADOS, total	15±4.1	—	—	—
ADOS, communication	4±2.5	—	—	—
ADOS, social	15±3.7	—	—	—
SCQ	12±4.3	—	—	—
GMDS, performance	85±24.2	107.1±15.2	*t* (30)=4.54	<0.000
CBCL, total	54±11.3	45.1±8.6	*t* (30)=2.35	<0.000
CBCL, internalizing	56±11.2	48.0±8.3	*t* (30)=3.87	<0.000
CBCL, externalizing	51±9.5	47.5±6	*t* (30)=3.43	<0.005

Abbreviations: ADOS, Autism Diagnostic Observation Schedule; ASD, autism spectrum disorder; CBCL, Child Behavior Check List; GMDS, Griffiths Mental Development Score; SCQ, Social Communication Questionnaire; TD, typical development.

**Table 2:** Transitions and fixations for the joint attention phase of the responding JA, initiating JA-1 and initiating JA-2 tasks

	*ASD*	*TD*	F*-value (*P*-value)*	*Effect size (*η*^2^)*
*Responding JA*				
Transitions face to target object^a^	1.82±1.59	1.73±1.67	0.03 (0.8)	0.001
Transitions face to non-target object^a^	1.52±1.18	2.00±1.36	3.3 (0.1)	0.05
Normalized transition score	0.07±0.58	0.05±0.61	0.51 (0.4)	0.06

*Initiating JA-1*				
Transitions target object to face^a^	3.31±2.57	1.60±1.63	2.42 (0.09)	0.10
Transitions non-target object to face^a^	0.50±0.52	1.40±1.18	5.89 (0.02)^b^	0.20
Normalized transition score^a^	0.36±0.25	0.10±0.13	5.73 (0.02)^b^	0.17
Transitions face to target object	3.86±2.50	1.93±1.33	6.23 (0.02)^b^	0.20
Transitions face to non-target object^a^	0.19±0.40	1.07±1.16	4.87 (0.03)	0.15
Between object transitions^a^	1.40±1.68	5.1±4.14	5.52 (0.02)^b^	0.2

*Initiating JA-2*				
Transitions target object to face^a^	3.62±2.63	1.71±1.37	7.41 (0.01)^b^	0.25
Transitions face to target object^a^	3.87±2.53	1.31±1.37	6.63 (0.008)^b^	0.25

Abbreviations: ASD, autism spectrum disorder; JA, joint attention; TD, typical development.

a Analysis performed on ranks.

b Significant after false discovery rate correction.
