# Supplementary material for: Disentangling the initiation from the response in joint attention: an eye-tracking study in toddlers with autism spectrum disorders
Source: Transl Psychiatry. 2016 May 17;6(5):e808–. doi: 10.1038/tp.2016.75 (PMC5070062; doi:10.1038/tp.2016.75)
Supplement: Supplementary Table S1 [file tp201675x2.docx]

*Table S1* Definitions of the joint attention measures for the three tasks (Responding JA, Initiating JA-1 and Initiating JA-2)

| Measure | Responding JA | Initiating JA-1 | Initiating JA-2 |
| --- | --- | --- | --- |
| Normalized accuracy | Gaze following: difference between frequency of first looks at target object and frequency of first looks at non-target object divided by the number of trials in which the child looks to either objects | Object following: difference between frequency of first looks to target object (moving object) and frequency of first looks to non-target object (still object) divided by the number of trials in which child looks to either objects | **_** |
| Transitions |  | | |
| face to target object (FTO) | Subject’s gaze shift from the face to target object | **_** | **_** |
| face to non-target object (FNTO) | Subject’s gaze shift from face to non-target object | **_** | **_** |
| target object to face (TOF) | **_** | Subject’s gaze shift from target object to face | Subject’s gaze shift from target object to face |
| non-target object to face (NTOF) | _ | Subject’s gaze shift from target object to face | **_** |
| between-objects transitions | **_** | Subject’s gaze shift from the target object to the non-target object and vice versa | **_** |
| Normalized transition score | Differences between transitions from face to target object and from face to non-target object divided by the total number of transitions from face to either object $\frac{FTO-FNTO}{FTO+FNTO}$  | Differences between transitions from target object to face and from non-target object to face divided by the total number of transitions from either object to face $\frac{TOF-NTOF}{TOF+NTOF}$  | **_** |
